# Assessing the implementation determinants of antimicrobial stewardship programmes in sub-Saharan Africa through the complexity lens. A CFIR-guided systematic review

**DOI:** 10.3389/fmicb.2025.1660778

**Published:** 2025-11-19

**Authors:** Thandizo Kapatsa, Akim Nelson Bwanali, Leonard Naphazi Kambewa, Vitumbiko Mkandawire, Gillian Mwale, Gracian Harawa, Stuart Ssebibubbu, Abdisalam Yusuf Ali, Steward Mudenda, Abigail Masi, Gertrude Chumbi, Chitemwa Moyo, Tumaini Makole, Jonathan S. Chung, SungJae Chung, Dowon Chung, Seongwon Chung, Victoria Hwang, Chloe Han, George Lee, Cynthia Chitule, Thomas Nyirenda, Adriano Focus Lubanga

**Affiliations:** 1 Education and Research, Clinical Research Education and Management Services (CREAMS), Lilongwe, Malawi; 2 Department of Laboratory Science, Dedza District Hospital, Ministry of Health, Dedza, Malawi; 3 Department of Obstetrics and Gynecology, Queen Elizabeth Central Hospital, Blantyre, Malawi; 4 National Antimicrobial Resistance Coordinating Center, Public Health Institute of Malawi, Lilongwe, Malawi; 5 Research Department, Afya na Haki Institute, Kampala, Uganda; 6 School of Public Health, Mount Kenya University, Thika, Kenya; 7 Department of Pharmacy, School of Health Sciences, University of Zambia, Lusaka, Zambia; 8 Pharmacy Council of Tanzania, Dar Es Salaam, Tanzania; 9 STEM Research Institute, Fairfax, VA, United States; 10 European and Developing Countries Clinical Trials Partnership (EDCTP), Strategic Partnerships and Capacity Development, Cape Town, South Africa; 11 Department of Global Health, Stellenbosch University, Cape Town, South Africa

**Keywords:** antimicrobial stewardship (AMS), implementation determinants, complexity science, CFIR framework, health systems, sub-Saharan Africa

## Abstract

**Background:**

Antimicrobial resistance (AMR) poses a significant threat in sub-Saharan Africa (SSA), where fragile health systems and under-resourced facilities exacerbate its burden. Antimicrobial stewardship (AMS) programs have been introduced as a key strategy to optimize antimicrobial use and curb AMR. However, the successful implementation of AMS in SSA remains limited. This systematic review assessed the implementation determinants of AMS programs in SSA using the Consolidated Framework for Implementation Research (CFIR).

**Methods:**

A systematic search was conducted across PubMed and Google Scholar for articles published between 2018 and 2024, following PRISMA guidelines. Studies were included if they reported on factors influencing AMS implementation in SSA. Data from 31 eligible studies were extracted and mapped according to the CFIR framework's five domains to identify key barriers and facilitators.

**Results:**

Major implementation barriers in SSA included underfunded health systems, limited diagnostic and laboratory infrastructure, lack of context-specific AMS guidelines, weak governance and policy enforcement, and insufficient training of healthcare providers. Enablers included hospital leadership support, stakeholder engagement, and existing global frameworks such as the WHO AWaRe guidelines. The review found poor integration of AMS into national health priorities and limited surveillance data, especially at the primary care level.

**Conclusion:**

AMS implementation in SSA is constrained by systemic, infrastructural, and educational challenges. Strengthening leadership, surveillance systems, healthcare worker training, and the development of context-specific AMS protocols are essential. Effective implementation will require tailored strategies grounded in local realities and supported by strong governance and sustainable funding mechanisms.

## Background

AMR is a top public health threat globally, claiming the lives over 1.27 million people annually (Iredell, 2019). Africa has the world's largest mortality rate from AMR infections, resulting in over 27 deaths per 100,000 (Africa CDC, 2024). It is estimated that the death toll from AMR could rise to as high as 10 million deaths annually by 2050 if the situation is left unchecked (Africa CDC, 2024). The fast rise in the incidence of AMR across African countries has led to the implementation of several strategies in the bid to curb its spread. Several studies have shown that measures to prevent infections, such as vaccinations and promoting hand hygiene, as well as improving hygiene in healthcare facilities, more than halves the risk of death and decrease the health burden of AMR (Horizon-europe, n.d.). Such AMS campaigns potentially reduce the burden of drug-resistant infections significantly, and in turn, reduce the morbidity and mortality associated with AMR (Baur et al., 2017).

As AMR continues to spread as far as to second- and third-line antimicrobial agents, the WHO has promoted a people-centered approach, a strategy that empowers individuals and communities to become AMR champions by improving knowledge and promoting responsible antimicrobial use (World Health Organization, 2023). It also supports AMS across all levels of care—community, primary, secondary, tertiary, and national—through practical interventions outlined in WHO's 2021 stewardship guide (WHO, 2022). These include clinician and public education, institutional treatment guidelines, antibiograms, and audit-feedback mechanisms, among others. Despite these tools, WHO acknowledges that local adaptation and implementation remain significant hurdles (WHO, 2022). According to WHO, antimicrobial stewardship (AMS) is defined as a systematic approach to educating and supporting healthcare professionals in following evidence-based guidelines for prescribing and administering antimicrobials (Toolkit AWHOP, 2019). This consists of integrated coherent actions which promotes the prudent use of antimicrobials to help improve patient outcomes across the continuum of care including prescribing antibiotics only when needed, selecting optimal drug regimens, drug dosing, route of administration, and duration of treatment following proper and optimized diagnosis. These actions, coupled with the implementation of infection prevention and control (IPC), enhancing water, sanitation and hygiene (WASH), and optimizing vaccination coverage, can significantly reduce the morbidity and mortality associated with AMR, particularly in developing countries (Lewnard et al., 2024).

To further curb the growing health and economic threat that is AMR, the World Health Organization (WHO) developed the Global Action Plan (GAP) to guide countries in creating comprehensive, inclusive strategies for AMR containment (OMS OM da S, 2017). The GAP emphasizes the continuity of successful treatment and prevention of infectious diseases with effective and safe medicines that are quality-assured, used responsibly, and accessible to all who need them. It also advocates for a continuous multi-sectoral approach to minimize the global impact of AMR and outlines important activities to be undertaken grounded in five key objectives: Improving awareness and understanding of AMR, Strengthening knowledge through surveillance and research, Reducing the incidence of infection, Optimizing the use of antimicrobial agents and Ensuring sustainable investment in countering AMR (OMS OM da S, 2017) at national level, National Action Plans (NAPs) serve as the main framework for country-level implementation of these goals. However, while they exist, their impact remains limited as many SSA countries struggle to translate these efforts into effective local implementation due to Inadequate funding, weak infrastructure, and limited local adaptation contribute to poor uptake of AMS interventions, especially at the primary and community levels (REVIVE, 2020). A report by the WHO in 2023 showed that only 28% (47 of 166 countries that reported for the AMR self-assessment survey) are implementing and monitoring their NAPs (World Health Organization, 2023). Understanding the contextual factors that shape AMS implementation is therefore essential for tailoring strategies that work in these resource-constrained settings.

To address this, implementation science offers useful frameworks for systematically identifying the barriers and facilitators to AMS uptake. One widely used model is the Consolidated Framework for Implementation Research (CFIR), which evaluates complex contextual factors that influence intervention success. The CFIR evaluates contextual factors across 5 domains, with 48 constructs and 19 sub-constructs (Means et al., 2020). The five domains consist of the innovation, the inner setting, the outer setting, individuals, and the implementation process. The inner setting is the place where the intervention is implemented, and the outer setting is the environment. Individuals refer to the people involved in implementing the intervention, and finally, the implementation process comprises activities or strategies deployed to implement the intervention (Means et al., 2020). This provides a better framework to evaluate AMS programs within multiple settings. In SSA, reviews evaluating AMS programs utilizing principles of implementation science in SSA remain limited. This review, therefore, seeks to address that gap by determining the implementation determinants for AMS programs within SSA and provide a description of how these determinants are embedded in complex systems.

## Research question

What are the implementation determinants for AMS programs in SSA?

## Methods

This systematic review followed the Preferred Reporting Items for Systematic Reviews and Meta-analyses (Page et al., 2021) reporting checklist to ensure a thorough and transparent presentation of the research process and findings and ensure systematic addressing of all key aspects of the review.

## Search strategy

To guide the identification and selection of relevant studies, we applied the PCC (Population–Concept–Context) framework recommended by the Joanna Briggs Institute for systematic reviews of implementation determinants. This framework is summarized in Table 1.

**Table 1:** Research concept breakdown according to the PCC framework.

**Element**	**Description**
Population	Health facilities, healthcare workers, and other stakeholders involved in antimicrobial stewardship activities in SSA.
Concept	Implementation determinants including barriers, facilitators, and contextual factors of AMS programmes, including leadership, adherence to recommendations, diagnostic capacity, policy enforcement, funding, and training.
Context	All healthcare levels in SSA countries including community, primary, secondary, and tertiary—between January 2018 and December 2024.

A comprehensive search was conducted in PubMed and Google Scholar between 1 and 28 February 2025 to identify articles on factors associated with AMS programme implementation in SSA, in accordance with PRISMA guidelines.

Literature search strategies were constructed using medical subject headings (MeSH) and text words related to the study's outcomes. Search terms were developed along the following key words: “Antimicrobial resistance,” “Antimicrobial Stewardship programmes,” “determinants” “SSA,” “barriers,” “facilitators” and specific names of countries within SSA. The keywords were combined using Boolean operators and applied truncations where necessary. All the relevant papers' reference lists were also checked for additional articles that could be included in the review.

## Inclusion criteria

Eligible studies for this review included original quantitative studies, qualitative observational studies as well as interventional studies published in English from January 1st, 2018 to December 31st 2024 that examined factors influencing the implementation of AMS programmes at any healthcare level in SSA or reported empirical data related to AMS implementation. This time period was purposely established to ensure that the systematic review captured the most recent research studies on the factors associated with the implementation of AMS programs in SSA. This timeframe also follows the formal establishment of the Africa Centers for Disease Control and Prevention (Africa CDC) in 2017 and its subsequent leadership role in AMR control across the continent. Study eligibility was also based on the papers.

## Exclusion criteria

Non-research articles, duplicates, editorials, commentaries, non-English publications, study protocols, case reports, opinion pieces lacking original data or empirical evidence and studies published before 2018 and outside of the SSA region were excluded from this systematic review. This review also excluded studies on AMR not directly addressing any AMS implementation factors.

## Article quality assessment

The quality of each article was assessed using the Cochrane guidelines for assessing bias in observational studies. The appraisal was conducted independently by two reviewers. A simplified quality appraisal checklist adapted from Cochrane guidance for observational studies covering key domains such as study design, sampling strategy, outcome measurement, data reporting, and quality assurance was applied to all included studies to provide an overview of potential sources of bias. The appraisal was conducted independently by two reviewers conducted, and any discrepancies were resolved through discussion until consensus was reached.

## Data extraction and synthesis

Retrieved articles were first systematically screened for relevance by two reviewers who independently screened titles/abstracts, assessed full-text eligibility, and applied the quality appraisal checklist. Inter-rater agreement in Rayyan was 99%, indicating almost perfect concordance. Discrepancies were resolved by discussion, where the two reviewers re-examined the original full text together, compared their extracted fields, and discussed the rationale for their interpretations until consensus was reached. Relevant articles that met the inclusion criteria were included in the data extraction process. The two researchers' extracted data independently using a standardized data extraction form was developed in Microsoft Excel based on PRISMA recommendations and the Consolidated Framework for Implementation Research (CFIR) developed in Microsoft word. The extracted data covered the following common areas: description of AMS interventions, the implementation context and strategy, reported implementation determinants (barriers, enablers, contextual factors) and the implementation outcomes. After the data had been obtained, a detailed summary was produced, capturing the study characteristics and their respective implementation determinants identified. The Implementation determinants were coded and categorized according to the CFIR domains and construct.

## Results

**Figure 1:**
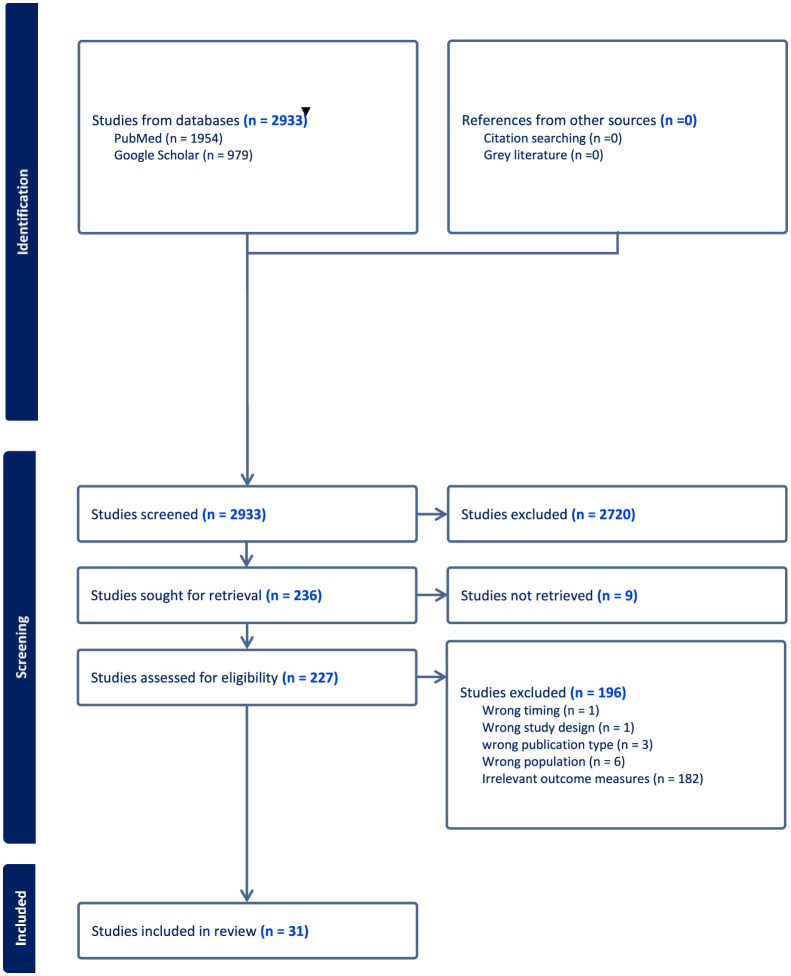
PRISMA summary of the systematic review process.

A total of 2,933 articles were initially identified through a comprehensive search of two primary databases: PubMed (1,954 articles) and Google Scholar (979 articles), as depicted in Figure 1. Following a rigorous screening process based on a pre-established inclusion criteria, 31 studies were selected and included in this review. Overall, of the 30 included studies, 4 were judged to be relatively high quality, 18 of moderate quality, and 9 of lower quality based on our adapted Cochrane checklist. The higher-quality studies tended to use mixed-methods, longitudinal, or cohort designs with clearer reporting, while most were observational cross-sectional or qualitative studies with limited methodological detail. Common weaknesses included small sample sizes, reliance on descriptive study designs, and insufficient reporting of study procedures. No studies were excluded on the basis of quality as the quality appraisal was used only to guide the interpretation of findings (Table 2).

**Table 2:** Study characteristics of included articles.

**Title**	**References**	**Author(s)**	**Publication year**	**Study design/setting**	**Study population**	**Implementation determinants identified**
Implementation of a Clinical decision support system for antimicrobial prescribing in sub-Saharan Africa: multisectoral qualitative study	Peiffer-Smadja et al., 2024	Nathan Peiffer-Smadja, Sophie Descousse, Elsa Courrèges et al.	2024	Qualitative approach through the use of semistructured interviews.	Stakeholders, health actors, scientists, physicians, biologists anthropologists, project managers, and funders with expertise in antimicrobial stewardship from 5 countries: Ivory Coast, Burkina Faso, Mali, Gabon, and Senegal	• Contextual factors influencing the implementation of eHealth tools existed at the individual, health care system, and national levels • At the individual level, the main challenge was to design a user-centered CDSS adapted to the prescriber's clinical routine and structural constraint • At the country level, weak pharmaceutical regulations, the lack of official guidelines for antimicrobial prescribing, limited access to clinical microbiology laboratories self-medication, and disparity in health care coverage lead to inappropriate antimicrobial use and could limit the implementation and diffusion of CDSS for antimicrobial prescribing • Participants emphasized the importance of building a solid eHealth ecosystem in their countries by establishing academic partnerships, developing physician networks, and involving diverse stakeholders to address challenges
Health care systems administrators perspectives on antimicrobial stewardship and infection prevention and control programs across three healthcare levels: a qualitative study	Aika and Enato, 2022	Isabel Naomi Aika, and Ehijie Enato	2022	Cross-sectional qualitative study	Health Care workers	• Major barriers are lack of management commitment and interprofessional rivalry which cuts across both secondary and tertiary facilities. Other barriers are shortage of professionals and poorly equipped laboratories
Implementation of antibiotic stewardship programmes in paediatric patients in regional referral hospitals in Tanzania: experience from prescribers and dispensers	Nkinda et al., 2022	Lilian Nkinda, Dorkasi L. Mwakawana, Upendo O. Kibwana et al.	2022	Exploratory qualitative study	Drug dispensers and Medical Doctors	• Barriers to the implementation of ASPs mentioned were lack of laboratory facilities to support culture and susceptibility tests, lack of materials and reagents, management pressure to prevent loss or to generate income, patients' influence and limited training opportunities. • Frequent stock out of drugs due to poor hospital management • Patient factors (demands) • Lack of continued medical Education with regard to AMR
Awareness and knowledge of antimicrobial resistance, antimicrobial stewardship and barriers to implementing antimicrobial susceptibility testing among medical laboratory scientists in Nigeria: a cross-sectional study	Huang and Eze, 2023	Sheng Huang and Ukpai A. Eze	2023	Descriptive cross-sectional study	Healthcare professionals registered as medical laboratory scientists	The high cost of testing, inadequate laboratory infrastructure, and a lack of skilled personnel are the major barriers to performing antibiotics susceptibility testing
Challenges of implementing antimicrobial stewardship tools in Low to Middle Income Countries (LMICs)	Fuller et al., 2022	Walter L. Fuller	2022	Cross-sectional study		• Lack of regulations or their enforcement on the prescriptions and sales of antimicrobials at the country or the regional level, the heterogeneity and complex nature of the healthcare system, poor clinical governance typified by the lack of AMS guidelines and poor adherence to such guidelines, when available, and the lack of national support for AMS in terms of human and financial resources and laboratory facilities • Only 8 (25.8%) out of the 31 countries have national AMS implementation plans or policies with defined goals, targets, and operational plans, which is a reflection of the country-level efforts prioritizing AMS as a core element for addressing the threat of AMR being low. • Level of AMS education is low among healthcare workers and in schools at the basic, primary, and secondary levels • Low laboratory diagnostic capacity in Africa due primarily to the lack of modern equipment and essential laboratory consumables
Challenges of implementing antimicrobial stewardship tools in Low to Middle Income Countries (LMICs)	Shamas et al., 2023	Nour Shamas, Elizabeth Stokle, Diane Ashiru-Oredope, Evelyn Wesangula	2023	Descriptive cross-sectional study		The fragility of states impedes the capacity to provide basic health services and mitigate the impact of infections and AMR. Although many governments have been attempting to improve access to healthcare through insurance schemes, over 92% of people in low-income countries and 73% in middle-income countries remain unin-sured [5]. Resources diverge from healthcare to basic need provision where AMS is often brushed aside. In such settings, even use of available AMS and IPC tools becomes challenging
Opportunities and barriers to implementing antibiotic stewardship in low and middle-income countries: lessons from a mixed-methods study in a tertiary care hospital	Gebretekle et al., 2018	Gebretekle et al.	2018	Mixed methods qualitative approach	Pharmacists and physicians	• Weak laboratory infrastructure was touted as a major obstacle for implementation of AMS. where almost all physicians did not send specimens or did not follow up on results because of delayed result reporting, and communication gaps between the laboratory and the treating teams • Most respondents admitted their antibiotic prescription behaviors are driven more by perceived risk of treatment failure and fear of retri-bution or academic punishment (especially for junior physicians), rather than evidence for infection
Implementation of an antimicrobial stewardship programme in three regional hospitals in the south-east of Liberia: lessons learn	Alabi et al., 2022	Alabi et al.	2022	Qualitative approach through the use of semistructured interviews.	One governmental hospital from River Gee, Grand Kru and Maryland respectively	• In general, the majority of the recommendations of the AMS team were followed (84.2%, 261/310, Table 2); only a small proportion of recommendations could not be followed due to non-availability of drugs • Senior hospital management leadership toward AMS (e.g., insufficient sustainable financial support), available expertise on infection management (e.g., limited access to experienced healthcare professionals), education and practical training (e.g., limited educational resources and regular training), monitoring and surveillance (e.g., compliance with one or more of the specific interventions, susceptibility rates and antimicrobial use are not regularly monitored) and reporting and feedback (e.g., reports on antibiotic susceptibility rates and antimicrobial use were not shared with prescribers).
Supply-chain factors and antimicrobial stewardship	Kamere et al., 2023	Kamere et al.	2023	Qualitative study	Supply chains from Kenya, Malawi, Nigeria, Sierra Leone, Uganda, United Republicof Tanzania and Zambia.	• Weak, underfunded health systems, failure to reimburse suppliers, unreliable supply chains and high out-of-pocket costs. This presents opportunities for substandard and falsified medicinal products to infiltrate the supply chain • Shortages and frequent stock-outs of essential medicines, including antimicrobials
Sustained reduction in third-generation cephalosporin usage in adult inpatients following introduction of an antimicrobial stewardship program in a large, urban hospital in Malawi	Lester et al., 2020	Lester et al.	2020	Qualitative approach through the use of semistructured interviews.	Healthcare workers	• Junior-level doctors, in particular, perceived that they now had a new resource that would guide them toward narrow-spectrum antibiotics, and valued the ability to access the guideline on their smart-phones. • Participants had profound trust in the information in the guidelines as they were locally appropriate with regard to antibiotic availability and pathogens. • Limited access to alternative antibiotics (quotes 4.1–4.3) and comprehensive diagnostics (quote 4.4), as well as an inadequate nursing capacity favoring antibiotics with once-daily dosing regimens. • Junior team members invariably took direction from senior colleagues on prescribing, and a rigid hierarchy frequently prohibited junior team members from challenging the prescribing of peers or senior colleagues
Antimicrobial stewardship programmes in Saudi hospitals: evidence from a national survey	Alghamdi et al., 2021	Alghamdi et al.	2021	Cross-sectional study	Ministry of health hospitals in Saudi-Arabia	• Around 85% of the respondents from the MOH hospitals reported that they do not have the knowledge and the technological resources needed to adopt and implement ASPs in their organization. Furthermore, only 36% of the respondents thought their organization has the financial resources needed as shown in Table 7. In relation to ASP teams/staff who would lead on the adoption and implementation of ASP, 82% of participants reported lack of necessary staff resources, and 77% reported lack of specific ASP staff/teams to champion the adoption and implementation of ASP in their organization • The majority of participants report not being pressured by the legislation to adopt and implement an ASP (73%) or to adhere to such legislation (83%)
Antimicrobial stewardship activities in public healthcare facilities in South Africa: a baseline for future direction	Engler et al., 2021	Engler et al.	2021	Cross-sectional study	Healthcare professionals from 26 public sector healthcare facilities across South Africa	• Entry level public healthcare facilities are hindered by many challenges to implement the Framework effectively due to their resource constraint which are higher than those of higher level facilities. • The limitations of infrastructure stretch far and wide, from too little space to keep patient files, affecting effective AMS, to too small or no space to do continuous education, to no handwashing soap and emollients, which also impedes IPC in general, subsequently having a negative impact on AMS. • Lack of support from those in managerial positions to effectively implement and adhere to guidelines
Knowledge, awareness and practice with antimicrobial stewardship programmes among healthcare providers in a Ghanaian tertiary hospital	Kpokiri et al., 2022	Kpokiri et al.	2021	Mixed methods study	Healthcare workers	• The healthcare providers across different specialties explained that they felt more confident carrying out AMS roles after an educational training on AMR and AMS. The nurses were competent in antibiotic drug administration and acted as patient advocates to question the antibiotics being prescribed. The pharmacists were also competent in delivering AMS role and providing information on medications for the rational prescription of antibiotics
Improving antimicrobial stewardship in the outpatient department of a district general hospital in Sierra Leone	Hamilton and Bugg, 2018	David Hamilton, Ian Bugg	2018	Cross-sectional study	Community health officers	• A 15 page guideline on antimicrobial prescribing was developed and made available to OPD community health officers and its effects were tracked. After 1 week with the guidelines, 139 patients were prescribed a total of 283 antimicrobials, of which 241 (85%) were appropriate when judged against the new local guidelines and 151 (53%) of those were also for the correct dose and course length. another tracking was done 2 months post guideline initialization and the progress had fallen to near baseline with 126 of 194 (65%) appropriate antimicrobial choices with 84 (43%) fully correct.
Antimicrobial resistance and antimicrobial stewardship in South Africa: a survey of healthcare workers in academic and non-academic hospitals	Reddy et al., 2023	Kessendri Reddy FCPath (SA) Micro et al.	2023	Cross-sectional survey	Doctors, nurses, and pharmacists	• A higher number of staff in academic health facilities—particularly nurses—felt that antimicrobial stewardship programs (ASPs) limit the decision-making freedom of prescribers and may negatively impact patient care. This perception might be influenced by the fact that academic settings in South Africa often have more experienced professionals who may be more protective of their clinical autonomy. • Almost a third of nurses in academic settings did not have time to further invest in ASPs and felt that only clinicians needed to understand AMS • Significantly fewer nurses felt that ASPs reduce antimicrobial use and can save costs, improve quality of care, reduce AMR and duration of hospital stay, impact nosocomial infection rates, and are currently needed.
Antimicrobial stewardship in private pharmacies in Wakiso district, Uganda: a qualitative study	Musoke et al., 2023	Musoke et al.	2023	Qualitative study	Private pharmacy staff	• According to the participants, the high cost of health-care was a major contributing factor to improper practices related to AMS. This is because many of the clients visited pharmacies without prescriptions, as they were avoiding the high consultation fees at private health facilities due to lack of money • Some participants mentioned that they needed to make sure their clients were satisfied with their services. In most cases, this meant pleasing them by giving them the antimicrobials requested, even if they were not appropriate • According to the participants, most of the AMS guidelines in Uganda were outdated and therefore not reliable. • Many of the participants felt that the implementation of AMS activities was difficult, as most pharmacies were in the business for profit. It was uncommon for a client to be denied an antibiotic if the client had money to purchase the drug.
Antibiotic use and stewardship indicators in the first- and second-level hospitals in Zambia: findings and implications for the future	Kalungia et al., 2022	Kalungia et al.	2022	Point-prevalence study	First and second level public hospitals	• Compliance with the Zambia National standard treatment guidelines was low at only 27.0% due to concerns about irregular updating of guidelines and if these are being adapted from high-income countries without local knowledge and input. Alongside this are issues around implementation and availability of STGs in the local hospitals,
Successful antibiotic stewardship in hospitalized children in a developing nation	Mustafa et al., 2020	Mustafa et al.	2020	Cross-sectional study	Medical records of children from 3 months to 13 years at the Department of Pediatrics at the Central Hospital, Pretoria, South Africa	• After a strict adherence to antibiotic prescription policy was implemented in September 2017, there was an immediate and noticeable improvement in limiting unnecessary and incorrect antibiotic prescriptions for children with obvious ‘viral' respiratory tract infection
Availability and use of therapeutic interchange policies in managing antimicrobial shortages among South African public sector hospitals; findings and implications	Chigome et al., 2020	Chigome et al.	2019	Quantitative descriptive study	Public hospital pharmacists	• The majority of participating hospitals had experienced antimicrobial shortages in the preceding 6 months that lasted for more than 40 days • Most pharmacists reported that they had resorted to dispensing later generation antimicrobials or more expensive alternatives when faced with shortage.
Status of antimicrobial stewardship programs in Nigerian tertiary healthcare facilities; findings and implications	Fadare et al., 2019	Fadare et al.	2019	Descriptive cross-sectional study	Public sector tertiary healthcare facilities	• None of the centers that participated in this study had any special salary or funding support for the members of their ASP teams.
Gaps and opportunities in sustainable medicines use in resource limited settings: a situational analysis of Uganda	Kamba et al., 2022	Kamba et al.	2022			• The veterinary health profession and medicines supply chain are weakly regulated and there is widespread medicines misuse and overuse which for antimicrobials fuels AMR • Overreliance on pharmaceutical imports leaving it vulnerable to supply chain disruptions
Implementation of a customized antimicrobial resistance laboratory scorecard in Cameroon, Ethiopia and Kenya	Trollip et al., 2022	Trollip et al.	2022	Pilot study	Laboratories in Cameroon, Ethiopia and Kenya	• Challenges to implementing the AMR Scorecard included lack of procurement of funding support for quality improvement and provision of cover for the trainers and mentors during programme-related absences. • Laboratories included in the pilot were not part of any active and ongoing programmes to improve laboratory quality such as the Strengthening Laboratory Management Toward Accreditation programm
Factors and outcomes related to the use of guideline-recommended antibiotics in patients with neutropenic fever at the Uganda Cancer Institute	Gulleen et al., 2021	Gulleen et al.	2021	Retrospective cohort study	Medical records of adults with acute leukemia admitted to Uganda Cancer Institute	• Most patients had non-guideline-recommended antibiotics ordered for initial NF treatment. • Clinicians tended to prescribe guideline recommended antibiotics to patients that presented severe symptoms of illness than those who had less threatening symptoms.
Antibiotic stories: a mixed-methods, multi-country analysis of household antibiotic use in Malawi, Uganda and Zimbabwe	Dixon et al., 2021	Dixon et al.	2021	Mixed-methods anthropological study	Rural and urban settings in Zimbabwe, Malawi and Uganda	• Use of private pharmacies and informal providers often forced by clinic stock outs • Increase in surveillance capacity in many LMICs to understand local use and resistance profiles, some of which is sensitive to socioeconomic and political factors driving high and rising antibiotic use and resistance which can positively inform AMS programs
Practices and motives behind antibiotics provision in drug outlets in Tanzania: a qualitative study	Ndaki et al., 2023	Ndaki et al.	2023	Qualitative study	Drug dispensers	• Dispensers were largely leb by their business interests and consumer pressures to dispense drugs rather than recommended guidelines
Perceptions and attitudes of health professionals on the establishment of an antimicrobial stewardship programme at a tertiary hospital in Gauteng province	Madeira, 2020	Hermenegilda Madeira	2020	Exploratory qualitative study	Healthcare workers	• Some respondents, some doctors lacked the knowledge needed to make a clinical judgement of whether antibiotics are indicated in certain situations • Sometimes antibiotics were given because it was the easier thing to as it avoids having to fully investigate the patient for sepsis. • Prolonged turnaround time for microbiological culture results to be received by the clinicians resulted in the prescription of antibiotics that may not have been appropriate • Doctors based their antibiotic prescribing on the existing practices that they observed when they first started working in the hospital rather than referring to prescription guidelines which proved difficult as Most of the participants reported that there were no hospital antibiotic prescribing guidelines or policies in place
Gamified antimicrobial decision support app (GADSA) changes antibiotics prescription behavior in surgeons in Nigeria: a hospital-based pilot study	Luedtke et al., 2023	Luedtke et al.	2023	Longitudinal pilot study	Surgeons	• There was a significant shift in guideline-concurrent AB type after receiving guidance from the virtual mentor as seen in scenarios where feedback from the app prompted surgeons to adjust their prescribing decision to become compliant with best-practice guideline emphasizing the success rates of AMS programs in the presence of continued and available mentorship and monitoring
Situational analysis of antimicrobial resistance, laboratory capacities, surveillance systems and containment activities in Ethiopia: a new and One Health approach	Beyene et al., 2023	Beyene et al.	2023	Situational analysis	Focal persons working in key institutions that have the potential to play roles in the prevention and AMR containment	• AMR surveillance activities have been challenged by the absence of sustainable investment, the absence of a budget to cover more sites in the country, and some laboratories lack the capacity to isolate pathogens and conduct an antimicrobial susceptibility test. • In the country, 60 human health establishments started the program, however, most of them are not active and didn't fulfill international standards.
Antimicrobial stewardship optimization in the emergency department: the effect of multiplex respiratory pathogen testing and targeted educational intervention	Durant et al., 2020	Durant et al.	2020	Cross-sectional study	Laboratory patient charts	• Providing timely results for influenza-based testing was a significant influencer of antibiotic prescription rate as patients were less likely to receive antibiotics if RPP-PCR results were available before the end of their visit to the ED or if the RPP-PCR result was positive for influenza.
Comparison of national antimicrobial treatment guidelines, African Union	Craig et al., 2022	Craig et al.	2022	Cross-sectional study	Websites of health ministries, national public health institutes or equivalent national government agenciesof all 55 AU Member States	• No guidelines were based on local disease burden or resistance profiles; possibly due to lack of national laboratory and surveillance capacities leading to gaps in the local evidence base • The existing guidelines did not include crucial information like recommended drug selection, dosage and duration of therapy varied, indicating a lack of clear, consensus clinical guidance and potential for the misuse of antimicrobials • Few guidelines incorporated antimicrobial stewardship principles, culture or antimicrobial susceptibility testing results into treatment recommendations
Handing out non-prescribed antibiotics is storing up trouble for the next generation!” Unpacking multitasker holder views of drivers and potential solutions in Ethiopia	Belachew et al., 2023	Sewunet Admasu Belachew, Lisa Hall and Linda A Selvey	2023	Phenomenological qualitative study	Pharmacy professionals	• Majority of participants emphasized that compliance with dispensing regulations and refusing to provide non-prescribed antibiotics would greatly impact the income of the CDRO • Majority of pharmacy professionals had inadequate awareness and inappropriate attitudes toward antibiotic use or provision, which contributes to the supply of antibiotics without prescription • Most pharmacists had confidence in diagnosing and treating various conditions without referring to guidelines • Lack of or a less rigorous enforcement of the regulation is a major factor for the dispensing of antibiotics without a prescription

The characteristics of these studies are presented in Table 1. Additionally, data extracted from the included studies were synthesized by two reviewers independently, according to the components of the Consolidated Framework for Implementation Research (CFIR): the outer and inner setting, the individual, the intervention characteristics, and the implementation process, as outlined in Table 3.

**Table 3:** CFIR-based analysis on implementation determinants of AMS in SSA.

**CFIR component**	**Context**
Intervention	Facilitators:
	- Implementation of contextualized AMS Interventions according to the type of hospital and available resources and structures.
	Barriers:
	- Lacking adaptation and contextualization of AMS guidelines which posed a barrier in under-resourced settings.
	- AMS programs being perceived as complex, especially in settings lacking trained personnel.
	- Weak supply chain systems and inadequate lab support
	- High cost of implementation (e.g., training, diagnostics, and staff time) especially in public hospitals.
Outer setting	Facilitators:
	- External funding or support from international partners.
	Barriers:
	- Inadequate diagnostic tools leading to empirical prescribing,
	- Inappropriate prescribing driven by patient expectations and pressure to receive antibiotics
	- Gaps in national policies and lack of enforcement mechanisms weakening implementation effectiveness
Inner setting	Facilitators:
	- Stable infrastructure to sustain AMS programs.
	- AMS being prioritized and championed by facility leadership
	Barriers:
	- Hierarchical cultures within healthcare settings often hindered junior staff from questioning their seniors in cases where they noted inappropriate prescriptions given.
	- Primary-level and rural facilities struggle to sustain AMS programs due to limited infrastructure.
Characteristics of individuals	Facilitators:
	- Facilities with continuous training and mentorship fostering gradual improvements in behavior change in prescribers' attitudes toward AMS
	Barriers:
	- Unsatisfactory health workers' awareness of AMR and AMS principles
	- Stewardship regarded as irrelevant by some health care workers, especially in resource-limited contexts.
	- Prescribers reliance on practices they observed when they first started working in the hospital, rather than referring to prescription guidelines.
Process	Facilitators:
	- Good stakeholder engagement and clear mapping of actions plans toward implementation of AMS frameworks.
	- Strong monitoring leading to improved compliance to interventions.
	- Presence of AMS Champions e.g. specialists and pharmacists.
	Barriers:
	- Poor coordination due to absence of designated AMS leads
	- Lack of follow-up and accountability resulting in inconsistent implementation execution.
	- Lack of formal mechanisms to evaluate AMS outcomes and progress.
	- Lack of action planning leading to stalled efforts.

The review identified several common implementation determinants affecting AMS in SSA. These included underfunded health systems, characterized by frequent stock-outs of essential antimicrobials and limited laboratory diagnostic capacity, which impairs the confirmation of infections and appropriate antimicrobial use. Furthermore, significant policy and regulatory gaps were observed, with only a few countries having functional AMS implementation plans and weak enforcement of pharmaceutical regulations resulting in widespread over-the-counter sales without prescriptions. The absence of digital health tools, reliance on outdated clinical guidelines, and lack of continuous professional development for healthcare workers were also noted as critical barriers to effective AMS implementation.

## Discussion

This systematic review aimed at assessing the implementation determinants of AMS programs in SSA using the consolidated framework for implementation research. The findings highlighted the underlying causes of limited progress in AMS implementations and sustenance among many countries in the sub-Saharan region, despite overwhelming evidence recognizing AMR as a global. The review further offers directions toward necessary targeted interventions that could be most effective to tackle AMR in the SSA context. These results align with existing literature in indicating the need for a comprehensive understanding of contextual, behavioral, technological, and sociocultural factors associated with AMS programs if they are to be successfully implemented and sustained.

### Resource constraints and health system fragility

Resource limitation emerged as one of the central barriers to AMS implementation. Many public health facilities in SSA are under-resourced, with budget allocations often favoring high-profile programs such as HIV/AIDS, malaria, and tuberculosis over AMS activities which are perceived as less significant (African Union, 2021). A study by Shamas et al. (2023) highlighted the fragility of health systems in LMICs that consequently impede the provision of basic services. In such settings, specialized programs like AMS are unlikely to receive attention or funding, as limited resources are typically directed toward more immediate healthcare needs (Shamas et al., 2023). Globally, the WHO has advocated for national AMS budgets, yet 74.2% of countries in the WHO African region lack dedicated AMS budget lines, and only 25.8% have national AMS implementation policies (Fuller et al., 2022). These findings suggest that strengthening health system governance and securing dedicated AMS funding by governments in low and middle-income countries is critical for the sustainable implementation of the activities.

### Limited diagnostic capabilities and laboratory infrastructure

Low to middle income countries face numerous challenges in implementing effective and sustainable AMR surveillance programs. These challenges include lack of infrastructural and institutional capacities, inadequate AMR surveillance, insufficient investment and human resources, underutilization of available data, and limited dissemination of such data to regulatory bodies. Viewed through a complexity lens, these challenges are not isolated obstacles but interrelated factors within health systems that interact to influence how AMS and AMR surveillance are implemented. Inadequacy of diagnostic microbiology services was found to be a major hindrance in most countries in SSA (Fuller et al., 2022; Lester et al., 2020). This often limits antimicrobial susceptibility testing, as the high cost of testing, inadequate laboratory infrastructure, and a lack of skilled personnel serve as major barriers ultimately promoting empirical prescribing (Huang and Eze, 2023) which runs counter to the goals of AMS. A study by Kamba et al. (2022) emphasized how poorly equipped laboratories, stockouts, and inadequate supply chains hinder effective AMS. Five of the 31 studies in this review also highlighted frequent shortages of essential medicines in public health facilities, which drove patients to private pharmacies and informal providers, undermining stewardship efforts (Dixon et al., 2021). Some hospitals had experienced these stock-outs, lasting over 40 days in the previous six months, prompting pharmacists to dispense later-generation or more expensive alternatives (Chigome et al., 2020). In some cases, a proportion of AMS recommendations could not be followed due to unavailability of the prescribed drugs (Alabi et al., 2022).

These issues stem from weak, underfunded health systems, failure to reimburse suppliers, unreliable supply chains, and high out-of-pocket costs which together create opportunities for substandard and falsified medicinal products to infiltrate the supply chain (Kamere et al., 2023). While the WHO recommends clinical bacteriology services at the primary hospital level, in practice, these are often limited to tertiary facilities and poorly integrated with clinical care (Jacobs et al., 2019). Strengthening bacteriological diagnostic capacity at primary healthcare facilities is essential, as these are often the first point of contact for a wide range of patients. Without access to proper diagnostic services at this level, patients are frequently treated empirically, increasing the risk of inappropriate antibiotic use and the spread of AMR (Lubanga et al., 2024). In ideal situations where rapid diagnostic test results are available in time, clinicians are less likely to prescribe antibiotics unnecessarily. This highlights how timely and accessible diagnostics can support more informed prescribing. However, such tools remain scarce across most settings in SSA (Durant et al., 2020). Additionally, significant data gaps persist regarding AMR in Africa, with estimates of over 40% of SSA countries lacking AMR data. This includesgaps on the true burden of AMR across humans, animals, and the environment, as well as on its transmission, evolution, and asymptomatic carriage (Shomuyiwa et al., 2022). This paucity of data frequently leads to insufficient treatment recommendations tailored to local circumstances.

### Lack of context specific AMS guidelines

Although AMS guidelines exist in various forms, their effectiveness is often undermined by poor adherence and a lack of contextual relevance. This review found that even where AMS guidelines were available, adherence remains suboptimal. Contributing factors include limited awareness, poor accessibility, and guidelines that are not well-aligned with local clinical realities (Wehking et al., 2024). In some instances, the available guidelines could not be used as they were outdated and therefore not reliable (Musoke et al., 2023). In a study by Kalungia et al. (2022), compliance to treatment guidelines was as low as 27.0% due to concerns about irregular updating of guidelines and their adaptation from high-income countries without local knowledge and input. Context-specific AMS guidelines, tailored to local antibiotic availability, have been shown to improve practitioners' adherence to recommended practices. In a Tanzanian study, antimicrobial surveillance was key to formulating locally relevant AMS protocols related to high *E. coli* resistance, particularly against trimethoprim-sulfamethoxazole and penicillin (Gentilotti et al., 2020). This allows for healthcare providers to optimize empiric therapy, reduce treatment failures, and preserve the effectiveness of remaining antibiotics by aligning prescribing practices with local resistance patterns. For example, locally tailored AMS guidelines in Sierra Leone was associated with improved adherence, underscoring how national context can shape implementation success (Hamilton and Bugg, 2018). Similarly, the WHO AWaRe antibiotic book offers concise, evidence-based guidance on selecting, dosing, and administering antibiotics for over 30 common infections in children and adults across primary and hospital care that can be adapted to local contexts (WHO, 2020). However, the development of such guidelines requires country-level surveillance data generated from adequate laboratories which scarce in SSA due to the absence of sustainable investment, the absence of a budget to cover more sites in the country, and lack the capacity to isolate pathogens and conduct an antimicrobial susceptibility tests (Beyene et al., 2023; Craig et al., 2022). Shomuyiwa et al. (2022) highlighted that over 40% of SSAn countries lack comprehensive AMR data, particularly on community-acquired infections, environmental reservoirs, and AMR mechanisms. These examples illustrate the complex interplay between surveillance capacity, data quality, and guideline adaptation as guidelines remain poorly contextualized without local data; yet poorly contextualized guidelines undermine stewardship and data collection, creating a reinforcing loop typical of complex systems. This highlights a critical gap in the region's ability to generate reliable data necessary for context-specific AMS guideline development, ultimately undermining efforts to improve antimicrobial use and curb resistance.

### Governance, regulation, and policy enforcement

Hospital leadership support plays a crucial role in AMS implementation through allocating resources, granting AMS teams the necessary authority, participating in AMS meetings, and helping resolve conflicts (Pauwels et al., 2025). Leadership, governance and regulatory enforcement act as interconnected, system-level factors that together shape the authority, resources and behavior of AMS teams across all levels of the health system. Weak governance and poor regulatory enforcement significantly hinder AMS practices as seen in a Saudi-Arabian study where over 70% of the participants reported not being pressured by the legislation to adopt and implement or to adhere to such legislation (Alghamdi et al., 2021). In the absence of managerial support, healthcare workers may lack the authority, resources, or motivation to implement AMS guidelines consistently and a perception that adherence is optional rather than essential. At the facility level, stewardship efforts require leadership commitment and clear accountability structures, yet major barriers such as lack of management commitment and inter-professional rivalry spanning both secondary and tertiary facilities undermine these efforts (Aika and Enato, 2022). Pollack and Srinivasan (2014) emphasized that hospital management plays a critical role in driving stewardship initiatives.

Among included studies, 10 studies specifically highlight the need for stronger regulatory frameworks to enforce appropriate antimicrobial use, alongside the integration of AMS into broader health governance structures such as establishing academic partnerships, developing physician networks, and involving diverse stakeholders to address challenges which are essential for building a solid e-Health ecosystems (Peiffer-Smadja et al., 2024). They also underscore a lack of managerial commitment and resource prioritization at both national and facility levels—key factors affecting the successful implementation and sustainability of AMS initiatives (Engler et al., 2021; Belachew et al., 2023). In the absence of strict regulatory oversight, drug dispensers may prioritize business interests and respond to consumer demand, frequently dispensing antibiotics contrary to established guidelines (Ndaki et al., 2023). This further calls for clear penalties to entities that contravene laws related to the sale of controlled antibiotics over the counter in drug stores and unregistered pharmacies. For example, In Saudi Arabia, the Ministry of Health enacted a nationwide antibiotic restriction policy in 2018, making it illegal to dispense antibiotics in private pharmacies without a valid prescription. This came with strict penalties including fines of up to 100,000 SAR, cancellation of pharmacy licensing, and even potential imprisonment. As a result, the proportion of pharmacies dispensing antibiotics without prescriptions dropped dramatically from nearly 97% to around 12%, according to simulated client studies (Bin Abdulhak et al., 2011). Similarly, the department of Pediatrics at the Central Hospital, Pretoria, South Africa saw an immediate and noticeable improvement in limiting unnecessary and incorrect antibiotic prescriptions for children with obvious viral respiratory tract infections after a strict adherence to an antibiotic prescription policy was implemented in September 2017 (Mustafa et al., 2020).

### Knowledge and training of healthcare providers

This review found widespread gaps in AMS knowledge among healthcare workers, rooted in poor awareness from basic through secondary education and worsened by limited ongoing training on AMR (Fuller et al., 2022; Nkinda et al., 2022). In some cases, prescribers lacked the knowledge needed to make a clinical judgment of whether antibiotics are indicated in certain situations (Madeira, 2020). A study among pharmacists and physicians revealed that most respondents' prescription behaviors were driven more by the perceived risk of treatment failure and fear of retribution or academic punishment rather than by clear clinical evidence of infection (Gebretekle et al., 2018). There was also a tendency among clinicians to prescribe guideline recommended antibiotics to patients that presented severe symptoms of illness than those who had less threatening symptoms (Gulleen et al., 2021). In some cases, prescribers undermined existing guidelines and based their antibiotic prescriptions on the existing practices that they observed when they first started working in the hospital rather than referring to prescription guidelines while others preferred to protect their clinical autonomy as they felt AMS programs limit their decision-making freedom and may negatively impact patient care (Reddy et al., 2023).

This clearly shows how AMS guidelines are undermined by healthcare workers even when they are readily available. For example, a study in Thailand reported that the stewardship team lacked the authority to directly intervene in prescribing practices; consequently, their recommendations were often ignored by individual clinicians (Anugulruengkitt et al., 2025). Our review also found that pre-service training on AMS was inconsistently incorporated into health professional curricula, with seven of the included studies calling for continuous professional development to keep practitioners updated on evolving AMR trends as they felt more confident carrying out AMS roles after an educational training on AMR and AMS (Kpokiri et al., 2022). There was however in some instances, a notable shift in guideline adherence among clinicians after receiving guidance from a mentor, illustrating that continued mentorship and monitoring can greatly improve compliance with best-practice AMS guidelines (Luedtke et al., 2023). These findings underscore the urgent need to institutionalize AMS training at both pre-service and in-service levels to ensure healthcare professionals are well-equipped to implement effective stewardship practices from the outset of their careers. This also clearly shows how governance gaps, prescriber behaviors, and training practices do not act in isolation but are inter-connected, making AMS implementation more complex especially in SSA contexts.

### Limited funding for AMS programs

A majority of the health care institutions in SSA are public, government owned facilities that are supported by national budgets. Health care financing is at the core of multiple implementation challenges noted in this study. As highlighted by Shamas et al. (2023), the fragility of LMICs impede capacity to provide basic health services where resources diverge from healthcare to basic need provision. Consequently, there is hardly any procurement of funding support for quality improvement, provision of cover for AMS trainers and mentors during programme-related absences, or special salary or funding support for members of their ASP teams. No Matches Found This was demonstrated repeatedly at national and facility levels in this review where resources diverge from healthcare to basic need provision where AMS is often brushed aside. In such settings, even use of available AMS and IPC tools becomes challenging as resources are diverged to other competing priorities such as acquisition of pharmaceuticals, tuberculosis and HIV/AIDS programs (Shamas et al., 2023). The Global Action Plan on AMR calls for LMIC's to allocate dedicated AMR funds toward implementation of AMR strategies, however there are low country efforts in the region as demonstrated by the lack of budget lines for AMS activities in over 70% of the 47 member states of the WHO African region and only 25.8% of the states having a national AMS implementation policy (WHO, 2021). Although national action plans on AMR are publicly available in some countries, with at least one individual responsible for AMR nationally, it appears that commitments on AMR made in the often-comprehensive NAPs are rarely met on time, exhibiting weak accountability for AMR results (Harant, 2022). This calls for strengthened governance of AMR and stewardship, raising an awareness of AMR among key stakeholders and the prioritization of AMR in the region amidst other competing priorities. At facility level, successful AMS relies on a strong commitment from senior hospital management leadership, accountability, and responsibilities, among other core elements (Pollack and Srinivasan, 2016).

## Study limitations

Our review used the CFIR to organize and synthesize findings from studies that did not use implementation science frameworks at any point. This required retrospective mapping of reported determinants onto CFIR domains, which may introduce interpretive bias and limit the precision of categorization of some of the identified themes. The review focused on SSA, and while regionally representative, some countries were underrepresented due to a limited published literature. Due to the use of the English language in our search strategy, some studies could have been missed with equally important findings from Francophone and Lusophone countries within SSA. This review relied on PubMed and Google Scholar for article retrieval and did not include gray literature which may have excluded relevant unpublished or non-indexed reports. In addition, the majority of included studies were observational and descriptive, with small sample sizes and limited methodological detail, which may affect the strength and generalizability of the findings.

## Future research directions

Future research should prioritize the use of other implementation science frameworks to evaluate AMS initiatives in SSA. Such approaches will help generate deeper insights into the contextual, behavioral, and organizational factors influencing program outcomes which will in the end improve the impact of AMS programs. Operational research focused on improving the uptake and adaptation of AMS guidelines in diverse healthcare settings is urgently needed, particularly in under-resourced rural and peri-urban environments. Further studies should also investigate community-level AMS interventions, such as school-based education, engagement with traditional healers, and public awareness campaigns, to better understand how grassroots strategies can complement institutional efforts. Future research should also link hospital AMS initiatives to wider One Health contexts since antibiotic use in community pharmacies, veterinary practice, agriculture and environmental factors are interconnected with hospital practices. Integrating these domains will be important for developing sustainable stewardship strategies. Future research should also explore AMS financing in low- and middle-income countries, focusing on public-private partnerships, donor support, and domestic funding to identify strategies for sustainable program implementation. There is also a need for longitudinal studies assessing the long-term impact of AMS implementation on prescribing behaviors and AMR trends. This will be critical in guiding policies and refining stewardship approaches across the region.

## Recommendations

To effectively address the implementation challenges of AMS programs in SSA, there is a critical need to institutionalize AMS training at both pre-service and in-service levels across all cadres of healthcare professionals. This will build foundational knowledge, promote adherence to guidelines, and foster a culture of rational antimicrobial use from the onset of clinical practice. Equally important is investing in diagnostic capacity, particularly at primary healthcare levels, to enable evidence-based prescribing and reduce the prevalent reliance on empirical treatment approaches, as highlighted in numerous studies. Countries should also prioritize the development of context-specific AMS guidelines informed by local resistance patterns and available resources that are accessible, practical, and adaptable to real-world clinical scenarios.

Leadership commitment, allocation of dedicated budget lines for AMS activities, and clear accountability frameworks is crucial to support program sustainability and integration into routine care. Several studies in this review highlighted that, without political and financial commitment, other AMS interventions struggled to take root or were short-lived. Additionally, policy enforcement must be intensified to curb inappropriate antimicrobial sales, particularly over-the-counter access in unregulated settings as evidence from included studies consistently showed widespread over the counter antibiotic misuse, underscoring the urgency of regulatory action. Regulatory bodies should actively monitor compliance and implement penalties where necessary. Finally, it is crucial to foster opportunities for sharing best practices, harmonizing data systems, and leveraging technical and financial support for AMS implementation regional through collaboration and partnerships with international organizations.

Alongside long-term changes, there is also need for immediate improvement measures in AMS implementation, including training healthcare professionals at both pre-service and in-service levels to enhance their knowledge and encourage rational prescribing. There is also need for AMS champions and mentorship programmes that will help motivate staff and improve adherence.

## Conclusion

This review highlights the multifaceted challenges impeding the effective implementation of AMS programs across SSA. While AMS is globally recognized as essential to combating AMR, its integration into fragile health systems is undermined by limited funding, poor diagnostic infrastructure, weak regulatory enforcement, and insufficient training of healthcare workers. Addressing these gaps requires dedicated investment, contextual adaptation of guidelines, and sustained political and institutional commitment. A complexity-informed, implementation science approach such as the CFIR proves critical for designing and evaluating stewardship programs that can thrive in diverse African contexts.

## Data Availability

The raw data supporting the conclusions of this article will be made available by the authors, without undue reservation.
